# Accidental cold-related injury leading to hospitalization in northern Sweden: an eight-year retrospective analysis

**DOI:** 10.1186/1757-7241-22-6

**Published:** 2014-01-27

**Authors:** Helge Brändström, Göran Johansson, Gordon G Giesbrecht, Karl-Axel Ängquist, Michael F Haney

**Affiliations:** 1 Department of Surgical and Perioperative Sciences, Anesthesia and Intensive Care Medicine, Faculty of Medicine, Umeå University, S-901 85 Umeå, Sweden; 2 Kinesiology and Recreation Management, and Anesthesia, University of Manitoba, Winnipeg, Canada; 3 Emergency and Disaster Medicine Centre, Umeå University, S-901 85 Umeå, Sweden

**Keywords:** Accidental hypothermia, Frostbite, Body temperature, Rewarming, Cold-water Drowning

## Abstract

**Background:**

Cold injuries are rare but important causes of hospitalization. We aimed to identify the magnitude of cold injury hospitalization, and assess causes, associated factors and treatment routines in a subarctic region.

**Methods:**

In this retrospective analysis of hospital records from the 4 northernmost counties in Sweden, cases from 2000-2007 were identified from the hospital registry by diagnosis codes for accidental hypothermia, frostbite, and cold-water drowning. Results were analyzed for pre-hospital site events, clinical events in-hospital, and complications observed with mild (temperature 34.9 - 32°C), moderate (31.9 - 28°C) and severe (<28°C), hypothermia as well as for frostbite and cold-water drowning.

**Results:**

From the 362 cases, average annual incidences for hypothermia, frostbite, and cold-water drowning were estimated to be 3.4/100 000, 1.5/100 000, and 0.8/100 000 inhabitants, respectively. Annual frequencies for hypothermia hospitalizations increased by approximately 3 cases/year during the study period. Twenty percent of the hypothermia cases were mild, 40% moderate, and 24% severe. For 12%, the lowest documented core temperature was 35°C or higher, for 4% there was no temperature documented. Body core temperature was seldom measured in pre-hospital locations. Of 362 cold injury admissions, 17 (5%) died in hospital related to their injuries. Associated co-factors and co-morbidities included ethanol consumption, dementia, and psychiatric diagnosis.

**Conclusions:**

The incidence of accidental hypothermia seems to be increasing in this studied sub-arctic region. Likely associated factors are recognized (ethanol intake, dementia, and psychiatric diagnosis).

## Background

Accidental cold injuries, including hypothermia, frostbite and cold-water drowning, are important causes of injury and hospitalization in subarctic regions [1-5]. Interest in these types of accidental injuries has increased after several cold-weather mass casualty events, [6,7] where immersion and submersion in cold water has led to life-threatening hypothermia and cold-water drowning. Incidences and outcomes of cold injury have been reported for some regions [8] or nations [9] and in military populations [10,11]. Accidental cold-related injury is a potentially life-threatening condition that can lead to significant morbidity and life-long effects. Populations in cold climate regions, without proper safeguards and preparation when outdoors, are always at risk for cold injury. Based on previously published material, it is recognized that the occurrence of cold-related injury is often multifactorial [12-16]. Factors contributing to fatal hypothermia have been recently described [17]. It is not known if cold injury hospitalization and in-hospital survival is increasing over time, since presumably there is steady progress in prevention and treatment.

The aim of this study was to identify the incidence of cold-related patient injuries, and describe the rescue-activities, pre- and in-hospital treatment, and injury panorama in Northern Sweden during an 8-year study period from 2000 to 2007. Additionally, we wanted to estimate the association between cold-related injuries and ethanol/drug use, psychiatric illness or dementia, and accidents.

## Methods

### Identification of the cohort

This retrospective study was conducted with approval from the Regional Ethical Review Board in Umeå, Sweden. Data was collected from patient records for those admitted to the 12 hospitals in northern Sweden, population approximately 900 000 (the northern four County Council health care districts) for eight years, from 2000 to 2007. Subjects were identified in the hospitals’ patient administrative system using the International Classification of Diseases, version 10 (ICD-10) [18]. The codes for hypothermia (T68), frostbite (T33.0-T35.7) and drowning (T75.1) were used as search criteria. We identified cold-water drowning based on drowning site water temperature < 20°C, [19,20] from municipality-reported lake/river temperatures at the time of the accident.

### Data collection from hospital records

The diagnosis of hypothermia was based on the history and setting, clinical signs and symptoms as interpreted by the hospital physician in charge of the patient, together with measurement of core temperature. The diagnosis of frostbite was based on the history or clinical setting, together with clinical signs either of being frostbitten and already thawed, or being frostbitten at admission. The diagnosis of drowning was based on history at admission by the attending physician.

Patient records were reviewed by the co-authors and dedicated research nurses, and a structured collection form was used to record data (data collection sheet in English provided as Additional file 1). Data was categorized into four main periods. First, at the accident scene: data on environmental conditions, clothing worn and type of exposure were recorded. Second, during pre-hospital transport: data on type(s) and duration(s) of transport(s), treatment and environmental conditions during transport were recorded. Third, during hospital admission: data on patient treatment, core temperature and cardio-respiratory status, location and severity of frostbite, medical history, alcohol/drug use/abuse, injury, laboratory findings from the initial injury period as well as discharge information and duration of hospital stay was recorded. Fourth, follow-up information: data on medical status, hypothermia or cold injury sequelae were recorded.

The site of temperature measurement was obtained when available. Mild hypothermia was defined as core temperature from 34.9 - 32°C. Moderate hypothermia was defined as core body temperature from 31.9 - 28°C. Severe hypothermia was defined as core temperature < 28°C [1,21,22]. Frostbite injuries were described by location and/or severity (i.e., superficial or deep). The diagnosis of psychiatric disease, dementia or somatic disease was based on the medical journal report of a previously established diagnosis or medical information from the patient’s family. Drowning was defined as those admitted alive with a history of submersion and who were given the hospitalization diagnosis T 75 according to ICD 10.

### Statistical methods

Concerning statistical analysis, univariate linear regression was performed for analysis of annual incidences from year to year, and R^2^ values were presented. A test for a single population proportions was performed to identify differences in frequencies in a single population (Z test, or paired difference test) [23]. A Pearson Chi Squared test was used to test for differences in distribution of frequencies. P-values less than 0.05 were considered significant in these tests.

## Results

### General incidences

**Figure 1:**
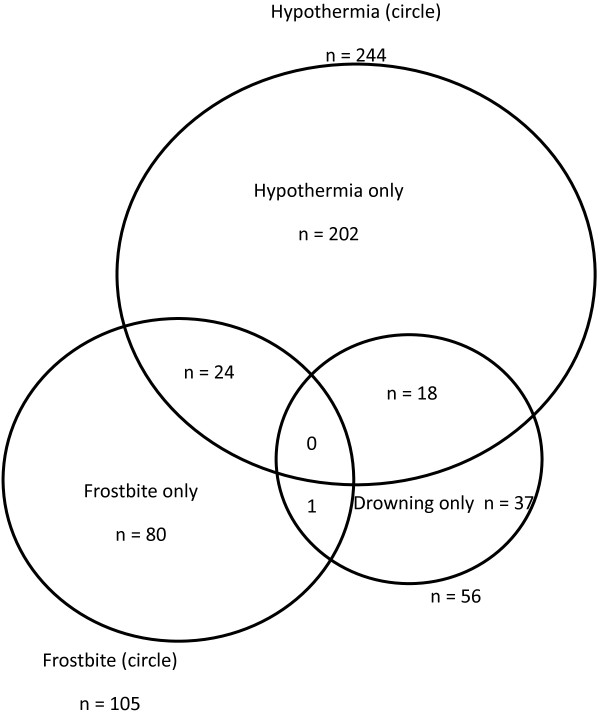
**Hypothermia, frostbite and drowning coincided in this cohort only in a minority of cases.** Note that for drowning cases, only 18/56 were documented to have become hypothermic. The frostbite cases are those severe enough to be treated in hospital, or where hypothermia was also present.

**Figure 2:**
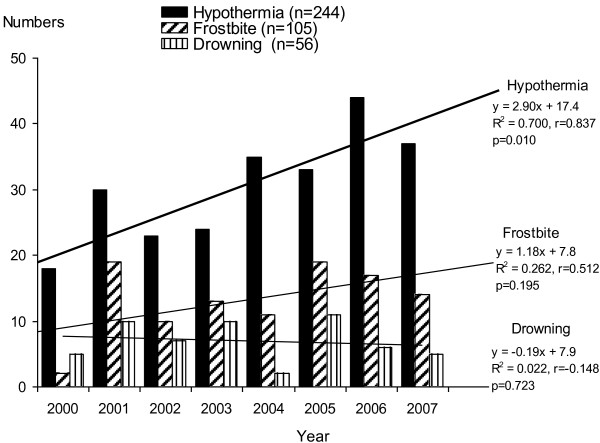
**Annual cold injury events are shown during the study period.** Linear regression analysis indicates that there was an increase in hypothermia annual frequency during this period, though not for the other diagnosis groups.

**Figure 3:**
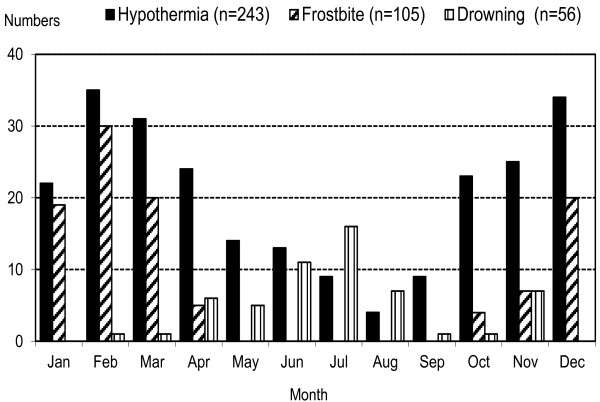
**Cold injury events by month, starting January, ending December.** The higher frequencies for hypothermia and frostbite are shown in the colder months (October through April, 194/244, 80%; p < 0.02), while outdoor drowning (water temperature < 20°C) is more prevalent in warmer months, May through September (Z test, p < 0.003).

There were 362 total cold-related injury patient admissions identified by hospital ICD 10 diagnosis codes during this period (2000-2007). This included 244 hypothermia, 105 frostbite, and 56 outdoor cold-water drowning cases. Twenty-two drownings were in very cold water (< 6°C, winter accident or fallen through ice) ([3]). This corresponded to an estimation of average regional annual incidence of hospitalization during this period for hypothermia of 3.4 per 100 000 residents, along with 1.5 frostbite cases/100 000, and 0.8 drowning cases per 100 000. Co-occurrence of hypothermia, frostbite and drowning was noted for some cases (Figure 1). The main finding was that there was a year-to-year increase in the incidence of hypothermia (p = 0.01) (Figure 2). Frostbite and cold-water drowning incidence did not change over the 8-year period of study. Cold injury events occurred more frequently in men (254, 70%) vs. women (108, 30%; p < 0.003) (Table 1). Time of year and location have an association with cold injury occurrence (Figure 3 and Table 2).

**Table 1:** Incidence (% of total) and median age (range) in each type of cold injury

	**Male**	**Female**	**All**
	**n=**	**n=**	**n=**
Hypothermia all	164(67%)*	80(33%)	244
Mild (34.9-32°C)	112(73%)	42(27%)	154
Moderate (28.0-31.9°C)	31(56%)	24(44%)	55
Severe (<28.0°C)	17(59%)	12(41%)	29
Missing data	4(67%)	2(33%)	6
Frostbite	82(78%)*	23(22%)	105
Cold water drowning	41(73%)*	15(27%)	56
**Age median (min-max)**			
Hypothermia all	57(8-94)	72(4-97)	60(4-97)
Mild (34.9-32°C)	56(8-94)	77(10-95)	59(8-95)
Moderate (28.0-31.9°C)	59(29-87)	77(4-94)	68(4-97)
Severe (<28.0°C)	66(15-83)	69(46-90)	66(15-90)
Frostbite	50(2-94)	55(2-98)	52(2-98)
Cold water drowning	42(1-83)	11(1-67)	36(1-83)

(*indicates larger proportion by Z test, p < 0.05).

**Table 2:** Site for accidental cold injury

	**n=**	**Indoor**	**Outdoor**	**Location not noted**
Hypothermia all	244	22(9%)	218(89%)	4
Mild (34.9-32°C)	154	11(7%)	140(91%)	3
Moderate (28.0-31.9°C)	55	8(15%)	46(84%)	1
Severe (<28.0°C)	29	3(10%)	26(90%)	
Frostbite	105	4(4%)	95(90%)	6
Cold water drowning	56	0	56(100%)	2

For the 244 hypothermia cases, core temperatures were seldom reported for pre-hospital locations (n = 30), but almost always at hospital (n = 233). The largest proportion of hospital patients had mild hypothermia (63%), with similar smaller proportions moderate (23%) or severe (19%). Temperatures were not noted in 11 (4%) patient journals despite the registered hospital admission diagnosis of hypothermia. Temperature measurement site was not noted in the hospital record for most patients (n = 225, 92%), and only a few were documented as rectal (5%) or tympanic membrane (15%) measurements (no esophageal temperature measurements were noted).

Hypothermia temperature categories and dysrhythmias are shown in Table 3, and only a few malignant dysrhythmias were reported, mostly for severe hypothermia patients. It is notable that an electrocardiogram was recorded and interpreted in only 43% of the hypothermia cases. Pre-injury activities for hypothermia, frostbite, and drowning were noted only sporadically in the medical records (Table 4). Laboratory findings at admission for hypothermia patients showed that pH, platelets, and potassium were distributed over a broad range (results not shown), with no clear patterns or trends discernible.

**Table 3:** Electrocardiographic findings

	**n=**	**ECG taken**	**ECG-AF**	**ECG-VES**	**ECG-VT**	**ECG-VF**
Hypothermia	244	104/244	19/104	4/104	1/104	2/104
Mild (34.9-32°C)	154	65/154	9/65	3/65	0/65	0/65
Moderate (28.0-31.9°C)	55	22/55	5/22	0/22	0/22	0/22
Severe (<28.0°C)	29	17/29	5/17	1/17	2/17	2/17
Missing data	6					
Frostbite	105	25/105	8/25	0/25	0/25	0/25
Cold water drowing	56	21/56	1/21	2/21	0/21	0/21

*Abbreviations:ECG* electrocardiogram, *AF* atrial fibrillation, *VES* frequent ventricular extrasystoles, *VT* ventricular tachycardia, *VF* ventricular fibrillation.

**Table 4:** Pre-injury activities, comorbidities, and cofactors

**All results in%**	**Hypothermia**	**Frostbite**	**Drowning**
*Activities*			
Car/driving	2	2	2
Snowmobile	5	9	4
Walking	5	20	0
Skiing	1	2	2
Mountain climbing	0	4	0
Fishing	3	0	2
Falling through ice	9	4	21
Outdoor skating	1	0	2
Not recorded	74	59	67
*Co-morbidities*			
Ischemic heart disease	20	10	5
Cerebrovascular disease	7	7	5
Multiple trauma	5	5	2
Psychiatric illness	7	14	2
Dementia	9	9	0
Epilepsy	2	0	4
Suicide attempts/apparent suicide	4	2	2
*Co-factors*			
Ethanol	34	28	30
Opiate	1	0	0
Benzodiazepine	5	5	4
Anti-depressive	1	1	0
Amphetamine	2	5	0

### Hospital management, rewarming

Once taken into hospital, active rewarming with a forced-air warming system was most commonly used. Often more than one warming method was employed at the same time (Table 5).

**Table 5:** Method of rewarming

		**Hypothermia severity**		
	**Hypothermia all**	**Mild**	**Moderate**	**Severe**
n=	244	154	55	29
Forced air warming	167	94	50	23
Warm bath	3	1	0	2
Warmed infusion	112	66	26	20
Extracorporeal	1	0	0	1
Water-filled garment	3	0	1	2
Periotoneal dialys	1	0	0	1
Radiant warming	6	5	0	1
Passive rewarming	40	38	2	0

### Frostbite

Deep frostbite was noted mostly on hands and feet in this hospitalized cohort (Table 6). Frostbite victims reported that they received their injuries with, among other activities, walking and snowmobiling. Associated factors included ethanol consumption (Table 4).

**Table 6:** Frostbite injuries

	**n**
*Depth of injury*	
Deep	65
Superficial	14
Both	24
Not specified	27
*Location of injury*	
One hand	20
Both hands	33
One foot	18
Both feet	45
Lower arm	3
Lower leg	14
Face	4
Thorax or abdomen	3

### Complications

For the total group of 362 admitted for cold injury, 16 (4%) died in-hospital at some point; all deaths but one were admitted with hypothermia. For hypothermia specifically, the in-hospital mortality was 6% (15/244). Cause of death for 15/16 was cardiac arrest, and 1/16 stroke. One patient admitted to hospital with mild hypothermia, died after many days in hospital related to chronic cardiovascular illness.

Of the total number that were admitted with frostbite (n = 105), amputation of the injured aspect resulted for a small number (n = 11, 10%), providing an average annual incidence for frostbite-related amputation of 0.15/100 000/year. Long-term physical impairment and musculoskeletal dysfunction as a result of frostbite injury was noted for 2/105 patients. Impaired sensorium after frostbite injury was noted for 3/105 patients. Cold intolerance or chronic pain was noted for 3/105 patients.

## Discussion

The main finding is an estimated recent incidence (3.4/100 000) for hypothermia with hospitalization which is higher than previously reported (1.1/100 000) from a similar region [9]. The findings for frostbite were confirmatory concerning annual incidence [24-26]. In our cohort, there were more male victims, while female victims had a more advanced age. The age and gender results could support the idea that older females may be more affected while having chronic illness, and that younger males may be more often victims of exposure due to daytime outdoor activities. Frostbite occurred at a higher rate among males, and could be related to more common outdoor occupational or leisure-time activities [26]. In-hospital mortality for hypothermia (6%) in this cohort was relatively low compared to a Dutch report [9].

Another notable finding was the increase in hypothermia incidence over the study period. This may be due to attitudes where community members under-appreciate the risks, and overestimate their own abilities and equipment. A second factor may be an increased number of community members at advanced age and with chronic illness, who are more vulnerable for cold injury. A possible confounding factor may be bias related to changes in physician awareness and increased reporting. There has been an active educational process in the region (and nationally, in Sweden) concerning cold injury during the study period, sponsored by the Swedish National Board of Health and Welfare [5].

A small number of the hypothermia cases occurred indoors, [17] and typically these occur where there is an open door, or in an indoor site which is not heated or poorly heated. An explanation may be that there are demographic changes, more elderly and medically debilitated in the population living at home, and often alone, which may explain a higher relative risk in the regional population [27,28]. Without earlier reports of cold-related injury incidences from the same region, it is not possible to determine if cold related injury incidences are changing, but a higher incidence of hypothermia than reported elsewhere suggests that it is possible.

Concerning pre-injury activities, the majority of victims had no specific activity identified in their patient journal. Possible explanations include that some of these victims were intoxicated (approximately 40% of total cases). Intoxication impairs the ability of an individual to assess and judge risks. It is also well recognized that many individuals combine intoxication with leisure outdoor activities such as snowmobile driving or fishing.

For a small subset of patients admitted with the diagnosis hypothermia, the only recorded temperature measurement was one that was over 35°C. There are several possible interpretations of this finding. One is that the clinicians simply failed to record a lower measured temperature. Another is that they measured a body core temperature correctly and the temperature was actually above 35°C at hospital admission. These types of patients, with mild cold injury are probably quite common, even though they usually are not identified with a formal hypothermia diagnosis in the national patient diagnosis registry.

A large portion of the hypothermia victims were diagnosed presumptively at the accident site, though a core temperature measurement was not recorded for this in the hospital records. One possible explanation of this is that the personnel had an opportunity to measure temperature, but did not prioritize doing this (and recording their measurements). Another possible explanation is that they did not have the means to measure core temperature. Once in the hospital, temperature was measured more systematically, although it was infrequently recorded which body area, and with which device. The optimal way to measure core temperature is in the esophagus, and this can be performed even in awake patients [29,30]. Other minimally invasive means of assessing include urinary bladder (catheter) probes, or rectally. Measuring temperature in the ear by infrared methods is widely regarded as inaccurate, though a closed ear canal temperature measurement performed correctly is considered reliable even in a cold environment [31]. It is important that both site and time of measurement are recorded [32] so that a time course of rewarming can be reviewed and assessed. Furthermore, documentation in the hospital record according to an international standard for on-site evaluation and treatment, including level of consciousness and presence or absence of shivering, along with field treatment should be performed [33,34].

External, non-invasive warming methods such as forced-air warming have become a favored method, even for moderate and severe hypothermia victims [35-37]. Other more invasive rewarming methods persist, though in these observations at a very low incidence. There were very few cardiac arrest and extracorporeal rewarming events observed in this cohort. This may be due to very long transport distances to a single centralized thoracic surgery resource for the whole region. There is no clear evidence in our findings to suggest that less invasive methods are less effective.

Limitations of this retrospective chart review study design include primarily the risk for not being able to identify and collect all the relevant patient information from the four phases of care for cold injury victims. While ambulance and hospital records were reviewed, it is possible that not all relevant information was recorded in these journals, or that in the paper chart review process, some relevant information was recorded but not identified and abstracted. There were clear limitations concerning documentation of measurement of core temperature, and the time course of rewarming. Also, this type of relatively small sample size with retrospective analysis cannot be used to make precise estimates of risk. Prospective structured data collection and a formalized effective data capture in a registry will be needed in order to allow more precise and less bias analysis of cold injury and associated or contributing factors.

## Conclusions

The incidence of accidental hypothermia seems to be increasing in this studied sub-arctic region. Likely associated factors are recognized (ethanol intake, dementia, and psychiatric diagnosis). We have not been able to evaluate if the regional health care system that was examined has been optimally effective with treatment, since limitations in hospital patient documentation restricted analysis of this.

## Competing interests

There were no financial competing interests for any of the authors.

## Authors’ contributions

HB has participated in study design, data collection, data analysis, and manuscript writing. GJ has participated in study design, data analysis and manuscript writing. GG has participated in study design, data analysis and manuscript writing. KÄ has participated in study design, data analysis and manuscript writing. MF has participated in study design, data analysis and manuscript writing. All authors have read and approved the final manuscript.

## Supplementary Material

Additional file 1 Data collection sheet. Click here for file
