# Supplementary material for: Accidental cold-related injury leading to hospitalization in northern Sweden: an eight-year retrospective analysis
Source: Scand J Trauma Resusc Emerg Med. 2014 Jan 27;22:6. doi: 10.1186/1757-7241-22-6 (PMC4016575; doi:10.1186/1757-7241-22-6)
Supplement: Additional file 1 — Data collection sheet. [file 1757-7241-22-6-S1.doc]

**Diagnosis**:  **Hypothermia** ICD-diagnosis for T.68 for ICD-10 (1997-)

 **Frostbite** ICD-diagnosis for T.33.0-T35.7 for ICD-10 (1997-)

 **Drowning** ICD-diagnosis for T.75.1 for ICD-10 (1997-)

**give diagnosis**:_______________________________

Person-number:__________________ Name:_____________________________________

Address:____________________________________________________________________

Hospital:_____________________________

**Accident site information**

Date (year/month/day):_________________________ Time:_______________________

Found  outdoors, shere:_______________________________

 indoors, where (ex. room with open window)_________________________

How long was the person incapacitated before they were found?_______________________

Outdoor temp: air:__________^o^C if indoors:____________^o^C

water:________^o^C

Wind: __________m/s

precipitation: __________ mm Type (ex. rain, snow, wet snow):_________________

Clothing:____________________________________________________________________

Hat:_____________________________________________________________________

Gloves:___________________________________________________________________

Shoes:____________________________________________________________________

Life vest:_________________________________________________________________

***Status at accident site***

Core body temperature:_______________^o^C

Temp taken (where?):  oral  rectal  axillary  ear  other:____________

For frostbite:

Localization:

Area:

Previous medical condition:________________________________

Alcohol intoxication:  yes  no describe:______________________

Drug intox:  yes  no describe:______________________

Level of consciousness (GCS/RLS/describe): _____________________________________

Pulse: ________________

BP: ________________

Resp rate:____________

Pupils: ________________

Reflexes: ________________

Muscle activity:  shivering  stiff

Speech:  distinct  slurring  no speech

Coordination and balance:  normal

 defective, desc describe:_______________________________________

**TRANSPORT**

Date (year/month/day):_________________________ time:_______________________

Temperature i vehicle during transport, Time for transport

Sled ____________^o^C ___________ hours, minutes

Ambulance ___________ ^o^C ___________ hours, minutes

Helicopter ___________ ^o^C ___________ hours, minutes

Airplane ___________ ^o^C ___________ hours, minutes

Other ___________ ^o^C ___________ hours, minutes

Body core temperature when transport started: ___________ ^o^C where measured: ________

ended: ___________^o^C where measured? ______

***Status during transport***

For Frostbite:

Localization:

Area?:

Level of consciousness GCS/RLS/describe): _______________________________________

Pulse: ________________

BP: ________________

Resp rate:____________

Pupils: ________________

Reflexes: ________________

Muscle activity:  shivering  stiff

Speech:  distinct  slurring  no speech

Coordination and balance:  normal  defective, Describe: __________________

**Hospital/ward**

Hospital:___________________________________ Ward:_____________________

A) Status at arrival

When? Date:______________ Time:__________________

Core body temperature: oral: _________^o^C esophageal: : _________^o^C

axillary: _______^o^C ear: _________^o^C

rectal: : _______^o^C other/where: _________^o^C

Level of consciousness (GCS/RLS/describe): ______________________________________

Pulse: ________________

BP: ________________

Resp rate:____________

Pupils: ________________

Reflexes: ________________

Muscle activity:  shivering  stiff

Speech:  distinct  slurring  no speech

Coordination and balance:  normal  defective, Describe: __________________

Size of cold injury:

Describe depth:_____________________

Describe area/size:___________________

Other injuries/describe? _______________________________________________________

___________________________________________________________________________

**B) Laboratory results at admission**

EKG ______________________ platelets _____________________

Hb, leucocytes ______________________ PT _____________________

hematocrit ______________________ aPTT _____________________

sodium ______________________ B-glucose _____________________

potassium ______________________

Krea ______________________ Amylase _____________________

O2Hb Sat% ______________________ Ethanol _____________________

pO_2_ ______________________

pCO_2_ ______________________ other _____________________

pH ______________________

**C) X-ray findings**

Chest x-ray ______________________

Skeletal ______________________

CT ______________________

Other ______________________

**D) Rewarming**

*Hypothermia/drowning*

Core body temerature at start: _________^o^C measured by: _________________

At finish: _________^o^C measured by:_________________

*Rewarming method

 passive, describe _____________________________ rewarming time:______________

 active, describe ________________________________ rewarming time: ______________

*Cold injury/frostbite*:

Method, describe ________________________________ rewarming time: ______________

Flushing after __________________ min

Teknetium scintiagraphy:  yes  no findings:_________________________

 Heart, describe (ex. arythmias, VF):___________________________________________

 Respiratory, describe (ex secretions, pulmonary edema)___________________________

 Neurological, describe (ex. seizures, deficits): ___________________________

 Bleeding, describe:_________________________________________________________

 Other, describe:_______________________________________________________

 Operations, describe:________________________________________________________

**E) Rewarmings end**

Core body temperature, where :____________________^o^C

Status when rewarming completed:

Level of consciousness (GCS/RLS/describe): ______________________________________

Pulse: ________________

BP: ________________

Resp rate:____________

Pupils: ________________

Reflexes: ________________

Muscle activity:  shivering  stiff

Speech:  distinct  slurring  no speech

Coordination and balance:  normal  defective, Describe: __________________

Extent of cold injury/frostbite:

Describe depth:________________

Desctrbe extent/area:_________________

Teknetium scintiagraphy:  yes  no findings:_________________________

 Heart, describe (ex. arythmias, VF):___________________________________________

 Respiratory, describe (ex secretions, pulmonary edema)___________________________

 Neurological, describe (ex. seizures, deficits): ___________________________

 Bleeding, describe:_________________________________________________________

 Other, describe:_______________________________________________________

 Operations/amputation, describe:_____________________________________________

**F) Discharged from hospital**

When (year/month/day):_________________

Season:_________________________

General status including: level of consciousness, neurological deficit, extent of cold injury/frostbite:_________________________________________________________

_________________________________________________________________________

**Follow up/return visit**

When? How long after injury (year/month/day):____________________________________

General status including: level of consciousness, neurological deficit, extent of cold injury/frostbite__________________________________________________________________________________________________________________________________________

Cold sensitivity, describe:_____________________________________________________

Impaired balance, describe:_____________________________________________________

Impaired walk, describe:_______________________________________________

Quality of life:______________________________________________________________

Other:_________________________________________________________________________________________________________________________________________________
